# Cost‐Effectiveness of Fracture Prevention in Postmenopausal Women With Early Breast Cancer in China

**DOI:** 10.1002/jcsm.70161

**Published:** 2025-12-10

**Authors:** Jin‐Yu Liu, Xi‐Han Lin, Yi‐Han Chen, Lin Wang, Ting Liu, Yu Zhang, Takahiro Mori, Kensuke Moriwaki, Ru‐Xu You

**Affiliations:** ^1^ Department of Pharmacy, Tongji Hospital, Tongji Medical College Huazhong University of Science and Technology Wuhan China; ^2^ Department of Pharmacy, Union Hospital, Tongji Medical College Huazhong University of Science and Technology Wuhan China; ^3^ Department of Pharmacy, the Second Xiangya Hospital Central South University Changsha China; ^4^ Health Services Research and Development Center University of Tsukuba Tsukuba Ibaraki Prefecture Japan; ^5^ Division of Health Policy and Management, Department of Biomedical Science, College of Life Sciences Ritsumeikan University Kusatsu Shiga Prefecture Japan

**Keywords:** aromatase inhibitors, breast cancer, cost‐effectiveness analysis, fracture prevention, osteoporosis

## Abstract

**Background:**

Aromatase inhibitors (AIs) significantly increase the risk of osteoporosis and related fractures in postmenopausal women with hormone receptor (HR)–positive early breast cancer (EBC), posing a substantial clinical and economic burden. Effective fracture prevention strategies are critical, especially in resource‐constrained settings such as China.

**Methods:**

A Markov microsimulation model was developed to evaluate the cost‐effectiveness of fracture prevention strategies for 60‐year‐old postmenopausal women with HR‐positive EBC treated with AIs in China. Six strategies were compared: (a) no intervention, (b) one‐time bone mineral density (BMD) screening followed by anti‐osteoporotic medication for patients with osteoporosis or osteopenia, (c) annual BMD screening followed by anti‐osteoporotic medication for patients with osteoporosis or osteopenia and (d) universal anti‐osteoporotic medication without prior BMD screening. Outcomes included incremental cost‐effectiveness ratios (ICERs) per quality‐adjusted life‐year (QALY) gained versus China's willingness‐to‐pay (WTP) threshold (3 × GDP/capita = $38 223/QALY).

**Results:**

All interventions reduced fractures but increased costs versus no intervention. For women aged 60–64 years, one‐time BMD screening followed by therapy for osteoporosis (*T*‐score ≤ −2.5) achieved an ICER of $17 368/QALY, below the WTP threshold. Annual screening yielded marginally higher QALYs (+0.0096) but higher costs (+$895.09), resulting in an ICER exceeding $38 223/QALY. For women ≥ 65 years, both one‐time screening for osteoporosis or osteopenia and universal therapy were cost‐effective. Universal therapy dominated in high‐risk subgroups (history of falls/fractures), achieving the highest QALY gains.

**Conclusions:**

One‐time BMD screening with selective alendronate for osteoporosis is cost‐effective for Chinese women aged ≥ 60 receiving AIs, aligning with China's healthcare constraints. Universal therapy becomes favourable for older or high‐risk subgroups. These data‐driven findings support tailored strategies to optimise bone health management and resource allocation.

## 1 Introduction

Aromatase inhibitors (AIs), such as Anastrozole, Letrozole and Exemestane, are the well‐known standard treatment for postmenopausal women with hormone receptor (HR)–positive early breast cancer (EBC), offering significant reductions in recurrence and improved survival outcomes [1, 2]. For example, the Arimidex, Tamoxifen, Alone or in Combination (ATAC) trial demonstrated that anastrozole reduced the risk of recurrence by 26% compared with tamoxifen in postmenopausal women [3]. However, despite their clinical benefits, AIs are associated with substantial risks, particularly concerning bone health. AIs therapy accelerates bone mineral density (BMD) loss, a well‐documented side effect distinct from the natural bone loss associated with menopause [4]. It has been reported that AIs therapy accelerates bone loss by approximately 10% per 5 years, a rate much higher than that seen in the general postmenopausal population [5, 6]. This accelerated bone loss significantly increases the risk of osteoporosis and fractures, particularly vertebral and hip fractures, which can lead to long‐term disability and even heightened mortality [7].

Current clinical guidelines from the American Society of Clinical Oncology (ASCO) and the National Comprehensive Cancer Network (NCCN) recommend BMD monitoring and the use of anti‐osteoporotic treatments such as bisphosphonates to mitigate these risks [8, 9, 10, 11]. It has been suggested that selective treatment with oral bisphosphonates, guided by annual BMD screening, is a cost‐effective approach in high‐income countries [12]. However, the economic feasibility of such strategies is unclear in resource‐limited settings, such as China, where healthcare resources are constrained. The costs of frequent BMD screenings and subsequent treatments could pose significant challenges, necessitating the exploration of more cost‐effective alternatives that balance patient outcomes with healthcare sustainability.

This study aims to assess the clinical and economic outcomes of various fracture prevention strategies for postmenopausal women with HR‐positive EBC treated with AIs in China. Specifically, it compares six strategies: (a) no intervention, (b) one‐time BMD screening followed by anti‐osteoporotic medication for patients with osteoporosis or osteopenia, (c) annual BMD screening followed by anti‐osteoporotic medication for osteoporosis or osteopenia and (d) universal anti‐osteoporotic medication without prior BMD screening. By comparing the outcomes of these strategies, this study intends to provide robust evidence on the most effective and cost‐efficient strategy for managing the bone health of this high‐risk population in a resource‐constrained healthcare system.

## 2 Methods

### 2.1 Study Design

This economic evaluation study adheres to the Consolidated Health Economic Evaluation Reporting Standards (CHEERS) statement (Supplementary Table S1) [13]. A Markov microsimulation model was developed from both the Chinese healthcare and society perspective, with a lifetime horizon, a one‐year cycle length, and a 5% annual discount rate applied to both health benefits and costs. The perspective of healthcare was selected as the base‐case analysis. The model was used to simulate the progression of HR‐positive EBC and the incidence of AI‐related fractures. A hypothetical cohort of 60‐year‐old women with HR‐positive EBC (stage I or II) was assumed to initiate a 5‐year therapy with AIs after completing radiation therapy. It was assumed that no patients in the base‐case cohort had previously received antiestrogen therapy or had a history of fragility fractures. The analysis was performed using TreeAge Pro 2019 (TreeAge Software Inc., Williamstown, MA, United States).

### 2.2 Strategies

**FIGURE 1:**
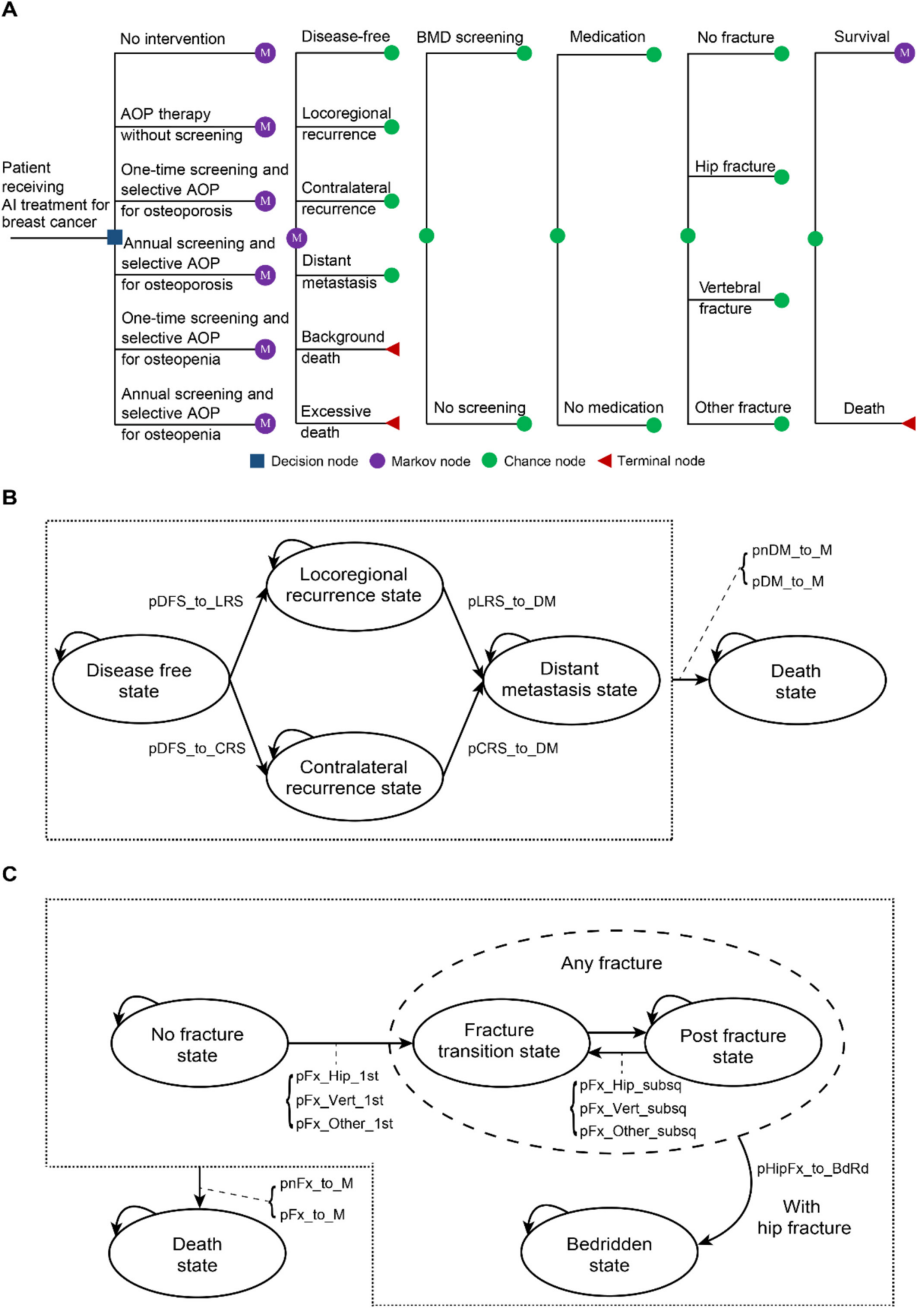
Schematic representation of model structure. (A) Markov transitions model schematic diagram. (A) Markov transition diagram summarising all health states. (B) Breast‐cancer transitions: Patients begin in the disease‐free state and may remain there or progress to locoregional or contralateral recurrence; patients in recurrence may progress to distant metastasis. (C) Fracture transitions: At any time, patients in any health state may experience a hip, vertebral or other fracture, which leads irreversibly to the post‐fracture state; hip fractures may result in a bedridden state. Patients with distant metastasis or post‐hip fracture experience higher mortality risk. AOP, antiresorptive therapy; BMD, bone mineral density; pCRS_to_DM, probability of transitioning from contralateral recurrence to distant metastasis; pDFS_to_CRS, probability of transitioning from disease‐free to contralateral recurrence; pDFS_to_LRS, probability of transitioning from disease‐free to locoregional recurrence; pDM_to_M, probability of death in the distant metastasis state; pFx_Hip_1st, pFx_Vert_1st, pFx_Other_1^st^, probabilities of a first hip, vertebral or other fracture; pFx_Hip_subsq, pFx_Vert_subsq, pFx_Other_subsq, probabilities of subsequent fractures; pFx_to_M, probability of death due to hip fracture; pHipFx_to_BdRd, probability of becoming bedridden following a hip fracture; pLRS_to_DM, probability of transitioning from locoregional recurrence to distant metastasis; pnDM_to_M, probability of death in states other than distant metastasis; pnFx_to_M, probability of death unrelated to hip fracture.

In Figure 1A, six strategies were compared:

- No intervention;
- One‐time dual‐energy X‐ray absorptiometry (DXA) screening: patients with osteoporosis (*T*‐score ≤ −2.5) or osteopenia (*T*‐score ≤ −1.0) received selective anti‐resorptive therapy after a single DXA test;
- Annual DXA screening with selective anti‐resorptive therapy for patients found to have osteoporosis or osteopenia;
- Universal anti‐resorptive therapy without DXA screening.

Oral alendronate (70 mg per week) was selected as the therapeutic agent based on current Chinese treatment guidelines [14]. As part of scenario analyses, we also examined biennial and quinquennial DXA screening as well as anti‐osteoporotic injections (denosumab and zoledronate).

### 2.3 Model

The model incorporated four breast‐cancer‐related Markov health states (Figure 1A): disease‐free, locoregional recurrence, contralateral recurrence and distant metastasis. As illustrated in Figure 1B, patients in the disease‐free state could remain in that state or transition to locoregional or contralateral recurrence. Patients with locoregional or contralateral recurrence could either remain in the same state or progress to distant metastasis; once a patient entered the distant metastasis state, they did not return to earlier cancer states.

Figure 1C describes fracture events within the model. Patients could experience a hip, vertebral or other fracture (pelvis, humerus, clavicle, hands/fingers, patella, tibia or fibula) at any time and in any health state. After a fracture, they transitioned irreversibly to the post‐fracture state. During each cycle, individuals without a prior fracture could suffer a simple fracture; each type of fracture could occur only once per cycle. A complex fracture could also occur as a combination of simple fractures. Patients who sustained a hip fracture could transition to a bedridden state, and no further fractures were modelled after entering that state. For individuals with a previous fracture, risks of subsequent fractures were multiplied by the relative risks of repeat fractures, except in the case of vertebral fractures.

It was assumed patients in the distant metastasis state or those who experienced a hip fracture faced higher mortality risks than those in other health states or those who sustained non‐hip fractures.

### 2.4 Input Parameters

The parameters used in this model are listed in Supplementary Tables S2 and S3. Several parameters, including BMD, fracture incidence, mortality rates and health utility values, were age‐dependent. The probabilities of progression in breast cancer were time‐dependent, while other parameters were assumed to be age‐independent.

#### 2.4.1 Progression of Breast cancer

Base‐case estimates for disease progression were derived from two key studies. The probabilities of remaining in a progression‐free state or transitioning to a progressive state were based on a meta‐analysis of randomised trials comparing 5 years of AIs versus 5 years of tamoxifen in women with HR‐positive EBC [15]. The probability of death due to distant metastasis was derived from a pooled analysis of phase 3 randomised trials involving cyclin‐dependent kinase 4/6 (CDK4/6) inhibitors plus endocrine therapy for patients with HR‐positive, advanced or metastatic breast cancer [16].

The main steps for deriving transition probabilities of cancer were data reconstruction based on Kaplan–Meier curves from the published studies to reconstruct individual patient data (IPD). Then, the IPD was used to performing parametric survival analysis to obtain the model parameters via selecting the best‐fitting parametric distribution (there are six common distributions to use, named exponential, Weibull, Gompertz, log‐logistic, log‐normal and generalised Gamma distributions), and the optimal distribution and its parameters were selected based on the criteria of Akaike information criterion and visual comparison of the simulated curve against original Kaplan–Meier curve. Take the parameters chosen in this study as an example, log‐normal distribution was used to compute survival function. In the given tth cycle, the survival probability was calculated as follow:

$$S\left(t\right)=1-\Phi \left(\frac{\ln\left(t\right)-\mu }{\sigma }\right)$$

where μ and σ are the parameters fitted from the simulated IPD data, Φ is the cumulative distribution function of the standard normal distribution. Finally, the transition probability from the survival function was calculated as follow:

$$p\left(t\right)=\frac{S\left(t\right)}{S\left(t-1\right)}$$

The analysis of deriving transition probabilities of cancer was performed using the R software (version 4.3.1).

#### 2.4.2 BMD and Fracture Incidence

Fracture incidences were predicted based on age and BMD‐specific values (Supplementary Table S3). Baseline BMD and changes in BMD during AIs therapy were estimated from published studies [17, 18]. It was assumed that a constant rate of BMD loss occurred during AIs therapy. Changes in BMD without AIs treatment were based on longitudinal results from the Study of Osteoporotic Fractures [18]. BMD values were converted into equivalent *Z*‐scores using age‐specific data from the third National Health and Nutrition Examination Survey (NHANES III) [19]. The probability of transitioning to a bedridden state following a hip fracture was sourced from a previous study conducted in Japan [20].

Transition probabilities p of fracture were calculated from the incidence rates r according to the following formula:

$$p=1-e^{-\mathrm{rt}}$$

where t means the fracture event occurring a time interval. Incidence rates of fragility fracture were listed in Supplementary Table S3. The equations for hip fracture, vertebral fracture and other fracture (e.g., wrist fracture or humeral fracture) were constructed by fitting curves on the basis of Chinese epidemiological data from published studies. The fit of the curves was determined by the *R*‐square and clinical plausibility. Then, the transition probabilities of fracture occurring over a time interval were calculated.

#### 2.4.3 Mortality

Mortality rates for each of the health states were calculated based on these background rates and the relative risk of death due to hip fractures or distant metastatic breast cancer. Background mortality rates were derived from the seventh national population census of China, published by the National Bureau of Statistics [21]. Excess mortality following hip fractures was taken from a population‐based cohort study of the Chinese population [22].

#### 2.4.4 Treatment Effect

The impact of treatment on fracture incidence was modelled under the assumption that women undergoing an anti‐osteoporotic therapy would reduce the relative risk of fragility fractures, regardless of their previous chemotherapy history. A post‐treatment residual effect of bisphosphonates (alendronate and zoledronate) was simulated for additional years and equivalent to the treatment period, gradually decaying over time. In the study, anti‐osteoporotic therapy was assumed to have no effect on the progression of breast cancer. Adherence to anti‐osteoporotic treatment was assumed to be consistent with published reports, and similar adherence rates were assumed for patients with breast cancer in China [23]. Meanwhile, two scenarios of adherence variances (72% and 54%) were considered in scenario analyses [23, 24, 25].

#### 2.4.5 Health State Utility Values

Health state utilities were obtained from Chinese population‐based studies that employed standardised methods. The utility for the general population was derived from EQ‐5D scores in the national health service survey [26]. Utilities for breast cancer–related health states were based on data from patients with breast cancer in China [27, 28]. The utility loss associated with each type of fracture was based on a meta‐analysis of fracture patients, and the utility for bedridden patients was taken from individuals receiving long‐term care [29, 30, 31].

#### 2.4.6 Costs

Osteoporosis‐related costs were assumed to be independent of breast cancer‐related costs, and breast cancer–related costs were not considered in this study. Osteoporosis‐related costs included drug costs (annual costs of alendronate, zoledronate, and denosumab), BMD screening costs, costs of fracture treatment and management annually (consisting of physician visits, inpatient treatment, laboratory tests and radiography, physical therapy, regular rehabilitation, etc.) and long‐term care costs associated with being bed ridden (shown in Supplementary Table S2). Drug costs and annual management costs for post‐fracture were obtained from the National Healthcare Security Administration of China [32]. The costs of BMD screening, fracture treatment and long‐term care costs were sourced from an economic burden analysis of fracture patients with osteoporosis in China [33]. All costs were inflated to 2022 prices using the Consumer Price Index (CPI) of China and converted to 2022 US dollars with an exchange rate of 1 USD = 6.7321 CNY [34].

In a perspective of society, indirect costs were considered. It was assumed that indirect costs indicated nonmedical attendant costs (e.g., transportation, special equipment, preventive herbal medicine), and the loss of out‐of‐work regarding unpaid caregivers, which is one of their children who are under 60 years old. The nonmedical costs were estimated based on statistical data published in China [33, 34, 35, 36].

### 2.5 Statistical Analysis

The base‐case analysis was conducted using a first‐order Monte Carlo simulation. Quality‐adjusted life years (QALYs) were used to measure health benefits. The incremental cost‐effectiveness ratio (ICER) for each strategy was calculated to compare the economic outcomes. The willingness‐to‐pay (WTP) threshold was set at three times the gross domestic product (GDP) per capita in China (USD $38 223/QALY in 2022). If a strategy had a higher ICER than the WTP, it was not considered cost‐effective.

Deterministic sensitivity analyses were performed to assess the robustness of model input parameters, with parameter ranges provided in Supplementary Table S2. Probabilistic sensitivity analysis was also conducted by randomly selecting values for each parameter from its assigned distribution. A second‐order Monte Carlo simulation with 1000 iterations was run to generate a cost‐effectiveness acceptability curve, showing the probability that each strategy was cost‐effective at varying WTP thresholds.

Scenario analyses were carried out with different combinations of (a) Age distribution: additional initiation age of 65–69, 70–74, and 75–79 years; (b) Risk factors: Fracture incidences were further adjusted based on patients' history of falls and prior fractures; (c) Screening intervals: In scenario analysis, BMD testing per 2 and 5 years was included; (d) Treatment adherence: Adherence decreased to 72% and 54% [23, 37]; (e) therapeutic drugs: denosumab (60 mg per 6 months, undergoing a 5‐year therapy) and zoledronate injection (5 mg annually, undergoing a 3‐year therapy) were considered; (f) the study perspective of society; and (g) a shorter time horizon set at 10 years.

## 3 Results

### 3.1 Model Validation

The 5‐year and 10‐year overall survival rates of the simulation cohort were 95.46% and 88.18%, respectively, which align closely with the results from the meta‐analysis (95.82% and 87.96%, respectively) [15]. Additionally, the incidence of any fracture within 10 years in the simulation cohort patients who received AIs treatment for 5 years was 14.48%, which is consistent with findings from a previous study (ranging from 12.23% to 14.2%) [15].

### 3.2 Base‐Case Analysis

**FIGURE 2:**
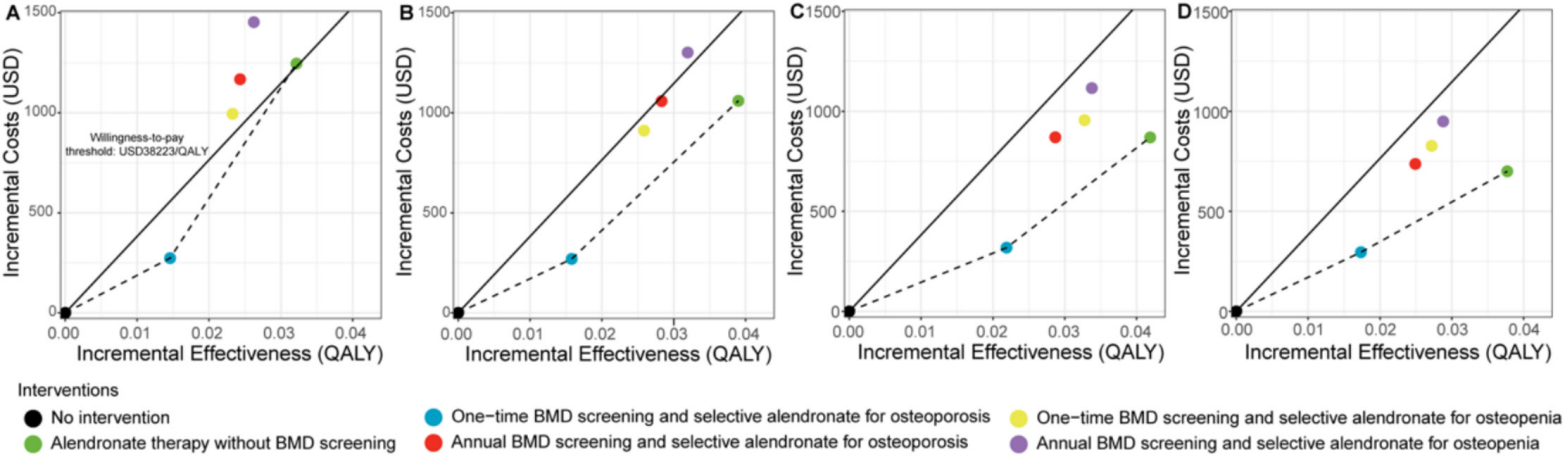
Cost‐effectiveness planes for all intervention strategies by initiation age: 60–64 (A), 65–69 (B), 70–74 (C) and 75–79 years (D). The dashed line represents the cost‐effectiveness frontier, while the solid line denotes the willingness‐to‐pay threshold. QALY, quality‐adjusted life years; USD, US dollars.

Figure 2 presents the cost‐effectiveness planes for all intervention strategies, stratified by initiation age. In detail, BMD screening combined with oral alendronate therapy effectively reduced the incidence of major osteoporotic fractures (hip and vertebral fractures), improved QALYs, but resulted in higher total treatment costs compared with the no‐intervention strategy (Supplementary Table S4). The ICERs for all strategies showed improved cost‐effectiveness as the initiation age increased.

For women aged 60–64 years, the most cost‐effective strategy was one‐time BMD screening followed by oral alendronate therapy for patients diagnosed with osteoporosis, with an ICER of $17 368 per QALY gained, which is below the WTP threshold of $38 223 per QALY. All other strategies in this age group were excluded, as their ICERs exceeded the WTP threshold. Although annual BMD screening resulted in a slightly higher QALY (incremental QALY: 0.0096), it was associated with an additional cost of $895.09. Expanding the treatment population to include patients with osteopenia also yielded a small increase in QALYs (incremental QALY: 0.0090 for one‐time screening) but incurred an additional cost of $722.91.

For women aged 65 years and older, both one‐time BMD screening for osteoporosis or osteopenia, and universal oral alendronate therapy were cost‐effective. Among these, one‐time BMD screening followed by oral alendronate therapy for patients with osteoporosis achieved the lowest ICERs across all age subgroups: $17 193 per QALY for women aged 65–69 years, $14 829 per QALY for those aged 70–74 years and $17 892 per QALY for women aged 75–79 years.

These findings suggest that the cost‐effectiveness of fracture prevention strategies improves with age, particularly when focusing on patients with osteoporosis. Furthermore, one‐time BMD screening followed by targeted therapy offers a viable and economical approach for older women treated with AIs.

### 3.3 Sensitivity Analysis

#### 3.3.1 Deterministic Sensitivity Analyses

**FIGURE 3:**
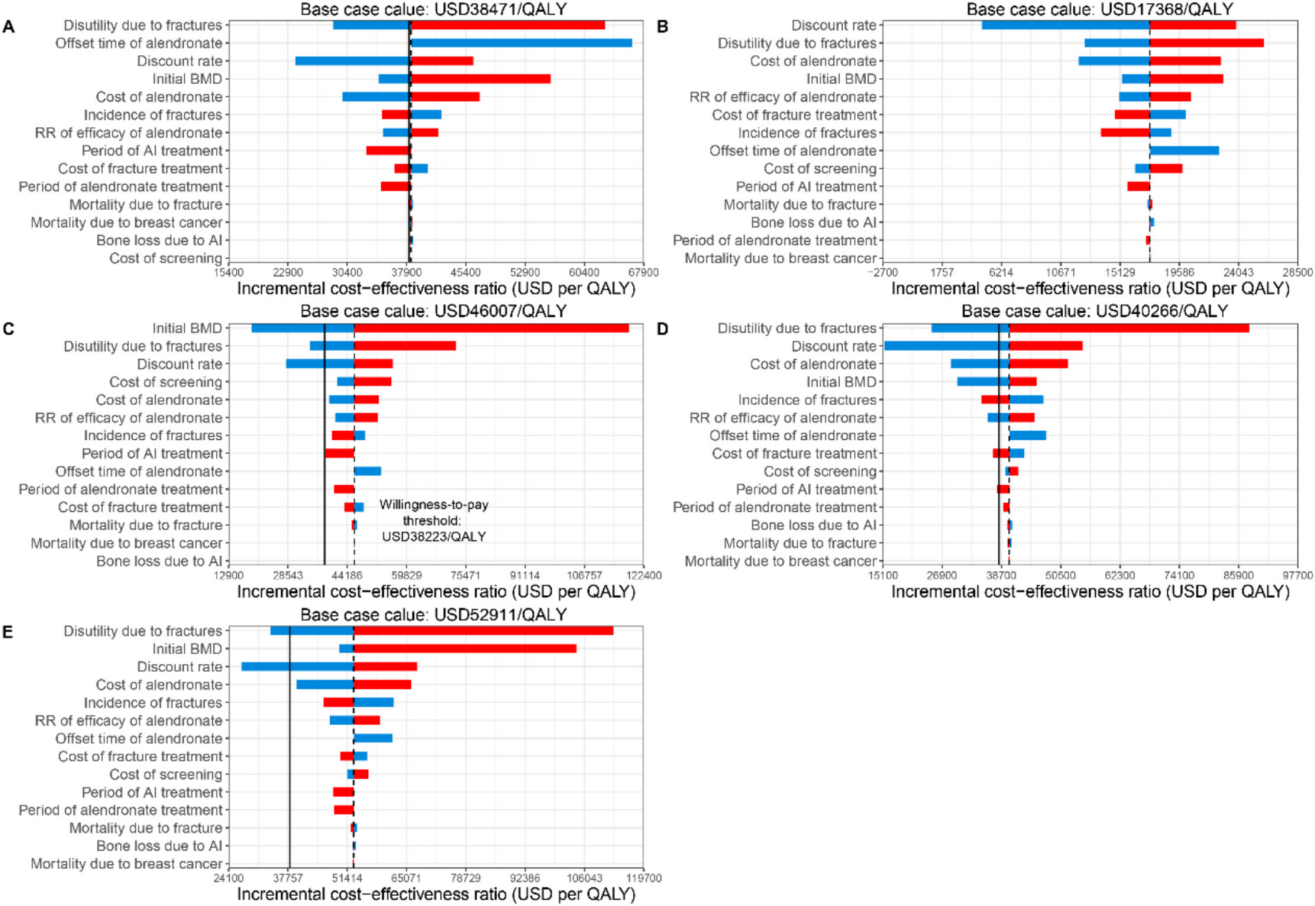
Deterministic sensitivity analysis results for (A) Alendronate therapy without BMD screening. (B) One‐time screening and selective alendronate for osteoporosis. (C) Annual screening and selective alendronate for osteoporosis. (D) One‐time screening and selective alendronate for osteopenia. (E) Annual screening and selective alendronate for osteopenia. The dashed line represents the base case ICER values, and the solid line indicates the WTP threshold. The blue bars show the impact of decreasing input parameters, while the red bars indicate the impact of increasing input parameters. QALY, quality‐adjusted life years; USD, US dollars.

The deterministic sensitivity analyses demonstrated the robustness of the base‐case findings, particularly for the strategy of one‐time BMD screening followed by oral alendronate therapy for patients with osteoporosis (Figure 3). Other strategies exhibited greater sensitivity to key parameters such as baseline BMD, the disutility associated with fractures and the discount rate, which had the potential to alter the base‐case conclusions.

#### 3.3.2 Probabilistic Sensitivity Analyses

**FIGURE 4:**
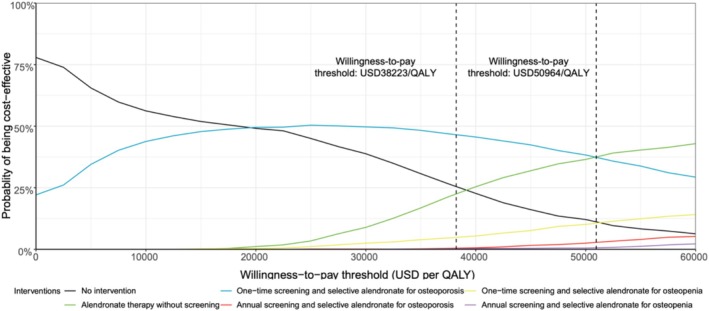
The cost‐effectiveness acceptability curve for all intervention strategies. The dashed line represents the willingness‐to‐pay threshold. QALY, quality‐adjusted life years; USD, US dollars.

The results of the probabilistic sensitivity analysis for the base‐case population are shown in the Figure 4. At a WTP threshold of $38 223 per QALY gained, the strategy of one‐time BMD screening followed by oral alendronate therapy for osteoporosis was deemed cost‐effective in 46.6% of simulations. However, when the WTP threshold was increased to $50 964 per QALY gained (equivalent to four times the GDP per capita), universal treatment with oral alendronate became the preferred strategy. These findings highlight the impact of varying WTP thresholds on the selection of the most cost‐effective intervention and emphasise the need to adjust fracture prevention strategies based on local economic conditions and policy priorities.

### 3.4 Scenario Analysis

**FIGURE 5:**
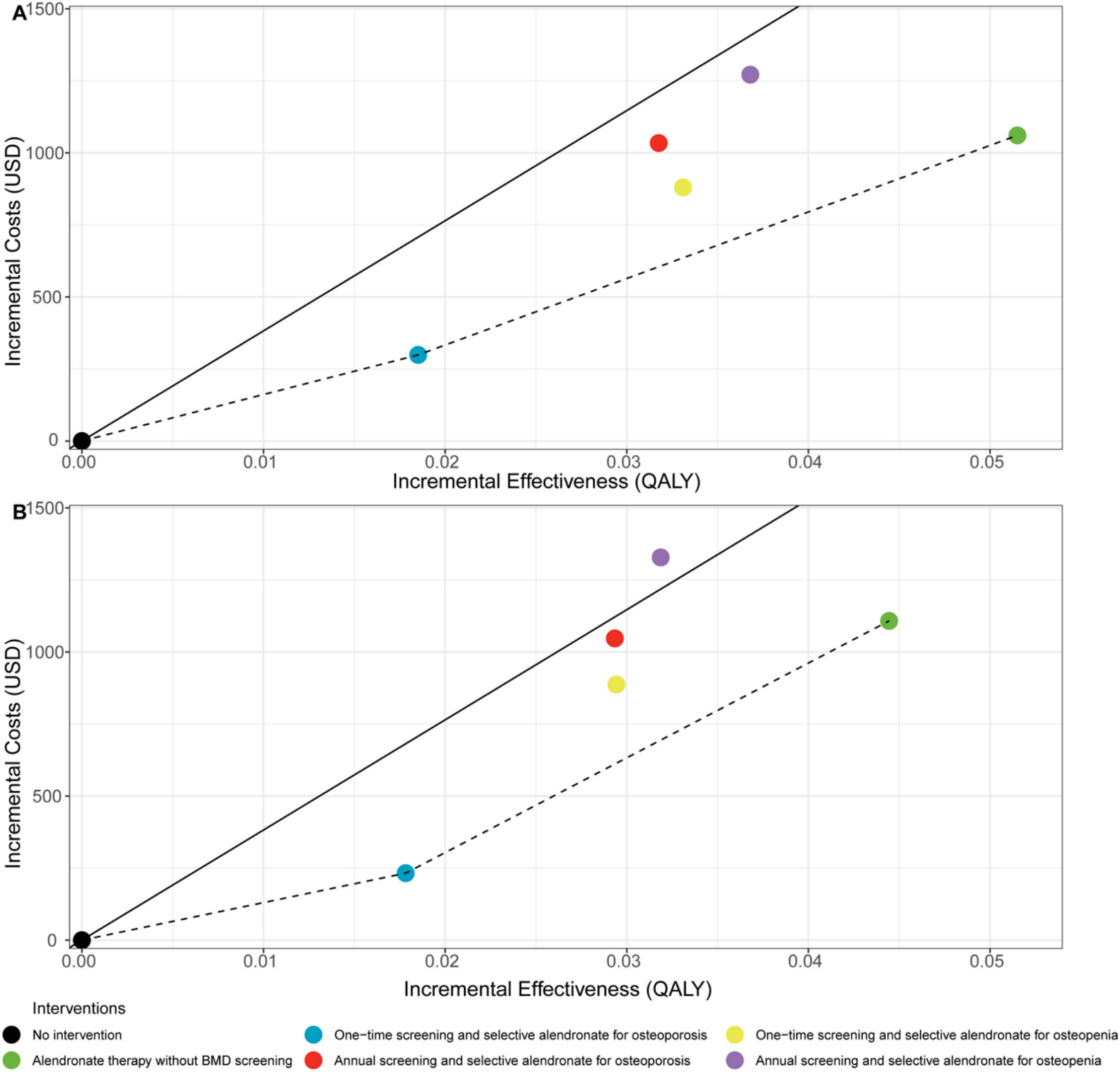
Cost‐effectiveness planes for all intervention strategies by scenario: history of prior fracture (A); history of fall (B). The dashed line represents the cost‐effectiveness frontier, and the solid line indicates the willingness‐to‐pay threshold. QALY, quality‐adjusted life years; USD, US dollars.

In patients with a history of fractures or falls, universal treatment with oral alendronate achieved the highest QALYs and was identified as the most cost‐effective strategy compared with other interventions, at a WTP threshold of $38 223 per QALY gained (Figure 5). Extending the screening interval decreases screening frequency and reduces costs (ranging from $26.17 per 2 years to $50.78 per 5 years) with slightly lower utilities, but ICERs exceeds the WTP threshold in China (Supplementary Table S5). The cost‐effectiveness of interventions is found to be associated with treatment adherence (shown in Supplementary Table S6), suggesting that the benefit of cost‐effectiveness is diminished when accompanied by low‐level adherence. When adherence decreased to 54%, the ICER of the one‐time screening for osteoporosis strategy increases significantly accompanied by an increase of $111.83 and a decrease of 0.0054 QALYs, resulting in an ICER value of $37 284/QALY and was close to the WTP threshold, indicating that lower adherence results in less cost‐effectiveness. In different therapeutic drug scenarios (shown in Supplementary Table S7), denosumab and zoledronate are cost‐effective under the WTP threshold, let alone in drug therapy without screening. Besides, zoledronate therapy shows the dominant cost‐effectiveness due to the decreased total costs and additional QALYs. After including indirect costs, one‐time screening for osteoporosis is still cost‐effective, although the ICER value increases to $29 193/QALY. Moreover, alendronate therapy becomes dominant and its ICER value is under the WTP threshold. In a shorter study horizon reduced to 10 years, ICERs of all interventions increase (shown in Supplementary Table S9). Of all strategies, one‐time screening for osteoporosis increases to $56 749 per QALY gained, not indicating cost‐effectiveness due to exceeding the WTP threshold of $38 223 per QALY.

## 4 Discussion

This base‐case analysis demonstrates that BMD screening combined with oral alendronate treatment is effective in reducing fracture incidence in China, although at a higher overall cost compared with no intervention. Specifically, for women aged 60–64 years, a single BMD screening followed by oral alendronate therapy for patients diagnosed with osteoporosis proves to be a cost‐effective strategy, with an ICER falling below the WTP threshold. For women aged 65 years and older, both BMD screening and oral alendronate therapy, when initiated after AIs treatment, are also identified as cost‐effective strategies. These findings emphasise the importance of targeted interventions for older populations, which are crucial for optimising healthcare resources in China, where there is a pressing need to balance clinical effectiveness with the constraints of the healthcare system.

The increased risk of osteoporosis and fractures associated with AIs therapy in breast cancer patients underscores the need for effective bone health management. AI‐induced bone loss accelerates fracture risk and contributes significantly to the healthcare burden [2]. This issue is especially pertinent in older women, who are at a higher risk for fractures, which can lead to long‐term disability or even increased mortality [38, 39]. Given the growing prevalence of AIs therapy and the aging population in China, effective pharmacological interventions are necessary to prevent secondary osteoporosis and its complications. Although pharmacological strategies are well established, there remains a lack of specific cost‐effectiveness analyses for this population in the Chinese healthcare context, highlighting the necessity for further research. This study is the first health economic evaluation of fracture prevention for Chinese women undergoing AI therapy, providing essential evidence to guide both clinical practice and policy decisions in the country.

Previous studies have demonstrated the cost‐effectiveness of regular BMD screening and anti‐osteoporosis treatment for postmenopausal women using AIs in high‐income countries. For example, Ito et al. found that annual BMD screening followed by selective anti‐osteoporosis therapy is cost‐effective in the United States, particularly for women aged 50–64 years [12]. Similarly, Sowa et al. reported favourable economic outcomes in Australia, although their study did not specifically address screening frequency [40]. However, in the present study, we found that although regular screening for BMD and subsequent treatment is clinically effective, the ICER value for annual screening exceeds the generally accepted cost‐effectiveness threshold in China, imposing a significant economic burden. This contrasts with the findings in the United States, where the economic context allows for more frequent screenings. Our findings suggest that a one‐time BMD screening followed by oral bisphosphonates treatment is a more cost‐effective approach, as it better aligns with the resource constraints and medical realities in China. This approach is more feasible and sustainable within China's healthcare system, where the demand for cost‐efficient interventions is high.

Furthermore, our study highlights the critical role of fracture history in determining the cost‐effectiveness of osteoporosis interventions. The burden of fractures on healthcare systems is particularly pronounced for older adults due to the increased risk of falls [41, 42, 43]. Previous economic evaluations have largely overlooked the impact of fracture history on cost‐effectiveness outcomes [20]. In contrast, our analysis fills this gap by demonstrating that one‐time BMD screening followed by oral alendronate therapy is particularly cost‐effective for patients with a history of fractures or falls. This underscores the importance of incorporating patient‐specific factors, such as fracture history, into cost‐effectiveness evaluations in order to design more personalised and efficient prevention strategies.

This study has several limitations that should be acknowledged. First, the measurement of BMD may not identify all patients with osteoporosis, as current screening techniques lack perfect accuracy. While this limitation could affect the precision of patient selection, its overall impact on the robustness of the cost‐effectiveness analysis is likely minimal. Second, although implementing screening with DXA as the current preferred standard test for diagnosed osteoporotic syndrome in people, on account of high expenditure and operation by professional technicians in China [44], DXA scanner resources are inadequate and available commonly in tertiary hospitals instead of primary healthcare. The lack of accessible healthcare resources for screening exists as a challenge. Future study would be conducted to evaluate DXA availability and cost‐effectiveness in relation to geographic and hospital‐tier disparities. Given the development disparities exist in China, the threshold of cost‐effectiveness may lack generalisability across various healthcare settings. Nevertheless, the economic benefits of fracture prevention strategies could be proved even in the least economically developed province in China at the WTP threshold of $20 052/QALY [34]. Third, while the majority of input parameters were based on data specific to the Chinese population, some were adapted from studies conducted in other Asian and non‐Asian countries. This may introduce minor variability, particularly when extrapolating findings to other regional contexts, and create some obstacles to policy development in the Chinese setting. Future research should aim to incorporate more comprehensive and localised data to further enhance the accuracy and generalisability of the results.

## 5 Conclusions

In conclusion, this study demonstrates that fracture prevention for breast cancer patients undergoing AI therapy, while increasing overall healthcare costs, remains a cost‐effective intervention in China. In detail, one‐time BMD screening followed by oral alendronate therapy for patients with osteoporosis at the initiation of AI treatment offers a highly economical strategy, including provinces with the lowest GDP per capita. This approach provides significant clinical and economic benefits, optimising healthcare resources in the context of China's unique medical and economic landscape. Our findings support the implementation of cost‐effective fracture prevention strategies tailored to local needs, contributing to improve patient outcomes and sustainable healthcare policies.

## Funding

This work was supported by the Talent Project established by the Chinese Pharmaceutical Association Hospital Pharmacy department (No. CPA‐Z05‐ZC‐2023‐003), the Hubei Provincial Natural Science Foundation Joint Fund for Innovation and Development Project (No. 2025AFD818) and the Specialized Research Program for Post‐market Clinical Research of Innovative Drugs (No. WKZX2023CX200001).

## Ethics Statement

Ethical approval was waived for the reason that this study is an economic model evaluation based on secondary analyses of research data.

## Conflicts of Interest

The authors declare no conflicts of interest.

## Supporting information




**Table S1:** Consolidated health economic reporting standards (CHEERS).
**Table S2:** Model parameters of transition probabilities, costs and utilities in Markov microsimulation.
**Table S3:** Equations for fragility fracture incidence and mortality hazard ratio after hip fracture.
**Table S4:** Intervention strategy outcomes by age.
**Table S5:** Outcomes of each intervention strategy including additional screening interval intervention at age 60–64.
**Table S6:** Outcomes of each intervention strategy in changing treatment adherence at age 60–64 years.
**Table S7:** Outcomes of each intervention strategy in changing therapeutic drugs at aged 60–64 years.
**Table S8:** Outcomes of each intervention strategy including indirect costs at age 60–64 years.
**Table S9:** Outcomes of each intervention strategy in 10‐year study horizon at age 60‐64 years.

## Data Availability

All data relevant to the study are included in the article and uploaded as Supporting Information.
