# Supplementary material for: Cost‐Effectiveness of Fracture Prevention in Postmenopausal Women With Early Breast Cancer in China
Source: J Cachexia Sarcopenia Muscle. 2025 Dec 10;16(6):e70161. doi: 10.1002/jcsm.70161 (PMC12690219; doi:10.1002/jcsm.70161)
Supplement: Supplementary file 1 — Table S1: Consolidated health economic reporting standards (CHEERS). Table S2: Model parameters of transition probabilities, costs and utilities in Markov microsimulation. Table S3: Equations for fragility fracture incidence and mortality hazard ratio after hip fracture. Table S4: Intervention strategy outcomes by age. Table S5: Outcomes of each intervention strategy including additional screening interval intervention at age 60–64. Table S6: Outcomes of each intervention strategy in changing treatment adherence at age 60–64 years. Table S7: Outcomes of each intervention strategy in changing therapeutic drugs at aged 60–64 years. Table S8: Outcomes of each intervention strategy including indirect costs at age 60–64 years. Table S9: Outcomes of each intervention strategy in 10‐year study horizon at age 60‐64 years. [file JCSM-16-e70161-s001.docx]

**Supplementary materials**

**Table S1** Consolidated health economic reporting standards (CHEERS)

| Topic | No. | Item | Location where item is reported |
| --- | --- | --- | --- |
| Title |  |  |  |
|  | 1 | Identify the study as an economic evaluation and specify the interventions being compared. | Title Page |
| Abstract |  |  |  |
|  | 2 | Provide a structured summary that highlights context, key methods, results, and alternative analyses. | Abstract |
| Introduction |  |  |  |
| Background and objectives | 3 | Give the context for the study, the study question, and its practical relevance for decision making in policy or practice. | Introduction |
| Methods |  |  |  |
| Health economic analysis plan | 4 | Indicate whether a health economic analysis plan was developed and where available. | Study Design, Paragraph 1 |
| Study population | 5 | Describe characteristics of the study population (such as age range, demographics, socioeconomic, or clinical characteristics). | Study Design, Paragraph 1 |
| Setting and location | 6 | Provide relevant contextual information that may influence findings. | Study Design, Paragraph 1 |
| Comparators | 7 | Describe the interventions or strategies being compared and why chosen. | Study Design, Paragraph 2 |
| Perspective | 8 | State the perspective(s) adopted by the study and why chosen. | Study Design, Paragraph 1 |
| Time horizon | 9 | State the time horizon for the study and why appropriate. | Study Design, Paragraph 1 |
| Discount rate | 10 | Report the discount rate(s) and reason chosen. | Study Design, Paragraph 1 |
| Selection of outcomes | 11 | Describe what outcomes were used as the measure(s) of benefit(s) and harm(s). | Statistical Analysis |
| Measurement of outcomes | 12 | Describe how outcomes used to capture benefit(s) and harm(s) were measured. | Input Parameters, Paragraph 9-10;  Statistical Analysis |
| Valuation of outcomes | 13 | Describe the population and methods used to measure and value outcomes. | Input Parameters, Paragraph 9-10 |
| Measurement and valuation of resources and costs | 14 | Describe how costs were valued. | Input Parameters, Paragraph 10 |
| Currency, price date, and conversion | 15 | Report the dates of the estimated resource quantities and unit costs, plus the currency and year of conversion. | Input Parameters, Paragraph 10 |
| Rationale and description of model | 16 | If modelling is used, describe in detail and why used. Report if the model is publicly available and where it can be accessed. | Input Parameters, Paragraph 3 |
| Analytics and assumptions | 17 | Describe any methods for analyzing or statistically transforming data, any extrapolation methods, and approaches for validating any model used. | Input Parameters, Paragraph 1-10; Model Validation |
| Characterizing heterogeneity | 18 | Describe any methods used for estimating how the results of the study vary for subgroups. | Statistical Analysis |
| Characterizing distributional effects | 19 | Describe how impacts are distributed across different individuals or adjustments made to reflect priority populations. | Statistical Analysis |
| Characterizing uncertainty | 20 | Describe methods to characterize any sources of uncertainty in the analysis. | Statistical Analysis |
| Approach to engagement with patients and others affected by the study | 21 | Describe any approaches to engage patients or service recipients, the general public, communities, or stakeholders (such as clinicians or payers) in the design of the study. | Not reported |
| Results |  |  |  |
| Study parameters | 22 | Report all analytic inputs (such as values, ranges, references) including uncertainty or distributional assumptions. | Supplementary materials, Table S2-S3 |
| Summary of main results | 23 | Report the mean values for the main categories of costs and outcomes of interest and summarize them in the most appropriate overall measure. | Results, Paragraph 1-2;  Supplementary materials, Table S4 |
| Effect of uncertainty | 24 | Describe how uncertainty about analytic judgments, inputs, or projections affect findings. Report the effect of choice of discount rate and time horizon, if applicable. | Results, Paragraph 3-4 |
| Effect of engagement with patients and others affected by the study | 25 | Report on any difference patient/service recipient, general public, community, or stakeholder involvement made to the approach or findings of the study | Not reported |
| Discussion |  |  |  |
| Study findings, limitations, generalizability, and current knowledge | 26 | Report key findings, limitations, ethical or equity considerations not captured, and how these could affect patients, policy, or practice. | Discussion |
| Other relevant information |  |  |  |
| Source of funding | 27 | Describe how the study was funded and any role of the funder in the identification, design, conduct, and reporting of the analysis | End of manuscript |
| Conflicts of interest | 28 | Report authors conflicts of interest according to journal or International Committee of Medical Journal Editors requirements. | End of manuscript |

**Table S2** Model parameters of transition probabilities, costs, and utilities in Markov microsimulation

| Variable | Value | Range | Distribution | Source |
| --- | --- | --- | --- | --- |
| Progression of breast cancer |  |  |  |  |
| Disease free to locoregional recurrence | μ=5.564446，σ=1.678216 | ±10% | Log-normal | [1] |
| Disease free to contralateral recurrence | μ=5.643153，σ=1.618009 | ±10% | Log-normal | [1] |
| Disease free to distant metastasis | μ=3.998037，σ=1.573521 | ±10% | Log-normal | [1] |
| Locoregional/Contralateral recurrence to distant metastasis | μ=3.752837，σ=1.478735 | ±10% | Log-normal | [1] |
| Distant metastasis to death | μ=1.145698，σ=1.598995 | ±10% | Log-normal | [2] |
| Treatment period of AI, years | 5 | 5-10 | Fixed |  |
| Mean BMD before AI, mean/SD(g/cm^2^) |  |  |  | [3] |
| 50-59 | 0.78 | 0.13 | Normal |  |
| 60-69 | 0.76 | 0.13 | Normal |  |
| 70-79 | 0.71 | 0.13 | Normal |  |
| 80- | 0.68 | 0.13 | Normal |  |
| Bone loss per year due to AI | ×0.0264 | ±10% | Fixed | [4] |
| Relative risk of hip fracture |  |  |  |  |
| With prior fragility fracture | 1.97 | 1.12-3.48 | Log-normal | [5] |
| With history of falls | 1.36 | 1.23-1.50 | Log-normal | [6] |
| Relative risk of vertebral fracture |  |  |  |  |
| With prior fragility fracture | 1.91 | 1.50-2.43 | Log-normal | [5] |
| With history of falls | 1.37 | 1.28-1.46 | Log-normal | [6] |
| Relative risk of other fracture |  |  |  |  |
| With prior fragility fracture | 1.91 | 1.50-2.43 | Log-normal | [5] |
| With history of falls | 1.41 | 1.32-1.51 | Log-normal | [6] |
| Probability of bed ridden after hip fracture | 0.136 | ±10% | Fixed | [7] |
| Efficacy of alendronate |  |  |  |  |
| Relative risk of hip fracture | 0.45 | 0.27-0.68 | Log-normal | [8] |
| Relative risk of vertebral fracture | 0.50 | 0.33-0.79 | Log-normal | [8] |
| Relative risk of other fracture | 0.78 | 0.66-0.92 | Log-normal | [8] |
| Treatment period, years | 5 | 5-10 | Fixed |  |
| Offset time, years | 5 | 0-5 | Fixed |  |
| Efficacy of denosumab |  |  |  |  |
| Relative risk of hip fracture | 0.50 | 0.27-0.86 | Log-normal | [8] |
| Relative risk of vertebral fracture | 0.33 | 0.19-0.65 | Log-normal | [8] |
| Relative risk of other fracture | 0.74 | 0.56-0.94 | Log-normal | [8] |
| Treatment period, years | 5 | 5-10 | Fixed |  |
| Offset time, years | 0 | - | - |  |
| Efficacy of zoledronate |  |  |  |  |
| Relative risk of hip fracture | 0.61 | 0.48-0.79 | Log-normal | [8] |
| Relative risk of vertebral fracture | 0.34 | 0.26-0.44 | Log-normal | [8] |
| Relative risk of other fracture | 0.78 | 0.66-0.92 | Log-normal | [8] |
| Treatment period, years | 3 | 3-6 | Fixed |  |
| Offset time, years | 3 | 0-3 | Fixed |  |
| Screening cost by DXA per year, US$ | 89.19 | ±20% |  | [9] |
| Annual drug cost of alendronate, US$ | 412.44 | ±20% | Triangular | [10] |
| Annual drug cost of  denosumab, US$ | 186.22 | ±20% | Triangular | [10] |
| Annual drug cost of zoledronate, US$ | 54.51 | ±20% | Triangular | [10] |
| Annual medical cost, US$ | 439.78 | ±20% | Triangular | [10] |
| Treatment costs of fracture per year, US$ |  |  |  | [9] |
| Hip fracture | 7,379.82 | ±20% | Triangular |  |
| Vertebral fracture | 1,361.12 | ±20% | Triangular |  |
| Other fracture | 1,758.30 | ±20% | Triangular |  |
| Annual cost of nursing care for bed ridden, US$ | 4,948.90 | ±20% | Triangular | [9] |
| Annual non-medical direct costs, US$ | ` |  |  | [9] |
| Hip fracture | 587.19 | ±20% | Triangular |  |
| Vertebral fracture | 458.56 | ±20% | Triangular |  |
| Other fracture | 327.97 | ±20% | Triangular |  |
| Annual loss of out-of-work costs for caregivers, US$ |  |  |  | [11–13] |
| Hip fracture | 516.67 | ±20% | Triangular |  |
| Vertebral fracture | 314.19 | ±20% | Triangular |  |
| Age-dependent event-free utility |  |  |  | [14] |
| 60-64 | 0.880 | 0.862-0.897 | Beta |  |
| 65-76 | 0.869 | 0.852-0.885 | Beta |  |
| 70-74 | 0.827 | 0.802-0.851 | Beta |  |
| 75-79 | 0.808 | 0.770-0.846 | Beta |  |
| 80-84 | 0.746 | 0.681-0.811 | Beta |  |
| 85+ | 0.707 | 0.561-0.853 | Beta |  |
| Disutility due to breast cancer, mean/SD |  |  |  |  |
| Disease free | ×0.86 | 0.17 | Beta | [15] |
| Locoregional/Contralateral recurrence |  |  |  | [15] |
| First year | ×0.72 | 0.16 | Beta |  |
| Subsequent years | ×0.85 | 0.16 | Beta |  |
| Distant metastasis |  |  |  | [16] |
| First year | ×0.43 | ±10% | Triangular |  |
| Subsequent years | ×0.62 | ±10% | Triangular |  |
| Disutility due to hip fracture |  |  |  | [17] |
| First year | ×0.776 | 0.720-0.844 | Beta |  |
| Subsequent years | ×0.855 | 0.800-0.909 | Beta |  |
| Disutility due to vertebral fracture |  |  |  | [17] |
| First year | ×0.724 | 0.667-0.779 | Beta |  |
| Subsequent years | ×0.868 | 0.827-0.922 | Beta |  |
| Disutility due to other fracture | ×0.910 | 0.880-0.940 | Beta | [18] |
| Utility for bed ridden | 0.192 | ±10% | Fixed | [19] |
| Discount rate | 0.05 | 0-0.08 | Fixed |  |

AI: aromatase inhibitors; BMD: bone mineral density.

**Table S3.** Equations for fragility fracture incidence and mortality hazard ratio after hip fracture

| Equation | Base case | Range | Reference |
| --- | --- | --- | --- |
| Equations for age- and BMD- dependent incidence rate of fracture |  |  |  |
| Hip fracture | $\frac{3.3889276976\cdot{10}^{-6}\cdot exp(0.085358\cdot Age)}{1.058239611}\cdot{1.4}^{-z}$ | ±30% | [20, 21] |
| Vertebral fracture without previous vertebral fracture | $\frac{6.990593537\cdot{10}^{-5}\cdot exp(0.067803\cdot Age)}{1.208314829}\cdot{1.85}^{-z}$ | ±30% | [22, 23] |
| Vertebral fracture with previous vertebral fracture | $\frac{5.64811735\cdot{10}^{-3}\cdot exp(0.033355\cdot Age)}{1.208314829}\cdot{1.85}^{-z}$ |  |  |
| Other fracture | $\frac{1.638631537\cdot{10}^{-5}\cdot exp(0.084641\cdot Age)}{1.058239611}\cdot{1.4}^{-z}$ | ±30% | [24, 25] |
| Hazard ratio of excess mortality after hip fracture | $2.6159-0.58523\cdot ln(Year after hip fracture)$ | ±30% | [26] |

Where $z$ is Z-score. The Z-score was calculated:

| $z= \frac{{BMD}_{current}-{BMD}_{age}}{SD}$ | (1) |
| --- | --- |

Where SD is standard deviation of BMD in individual of the same age, race and sex.${BMD}_{age}$ is the mean BMD in population of the same age, race and sex. ${BMD}_{current}$ is the BMD screened from the individual in the study. Based on Chinese epidemiological data, the values BMD could be fitted as follows:

| ${BMD}_{age}= 0.9980954- 0.004975\cdot Age$ | (2) |
| --- | --- |
| ${BMD}_{current}= {BMD}_{start\_age}- 0.004975\cdot Year\_from\_start-({BMD}_{start\_age} \times{BMD}_{loss by AI})\cdot Year\_from\_start$ | (3) |

**Table S4.** Intervention strategy outcomes by age

| **Interventions** | **10-year cumulative**  **fracture**  **incidence, %** | | |  |  |  |
| --- | --- | --- | --- | --- | --- | --- |
|  | **MOF** | | **All** | **Costs, $** | **QALYs** | **ICER, $/QALY** |
| *Age 60-64 years (base case)* |  |  | |  |  |  |
| No intervention | 11.92 | 17.00 | | 2832.43 | 8.2667 | Ref |
| Alendronate therapy without screening | 6.74 | 10.98 | | 4064.79 | 8.2987 | 38471 |
| One-time screening and selective alendronate for osteoporosis | 10.16 | 15.05 | | 3106.48 | 8.2824 | 17368 |
| Annual screening and selective alendronate for osteoporosis | 9.18 | 13.83 | | 4001.57 | 8.2921 | 46007 |
| One-time screening and selective alendronate for osteopenia | 8.98 | 13.42 | | 3829.39 | 8.2914 | 40266 |
| Annual screening and selective alendronate for osteopenia | 8.50 | 12.74 | | 4289.83 | 8.2942 | 52911 |
| *Age 65-69 year* |  |  | |  |  |  |
| No intervention | 17.83 | 25.58 | | 2963.30 | 7.0494 | Ref |
| Alendronate therapy without screening | 9.86 | 16.28 | | 4032.27 | 7.0887 | 27235 |
| One-time screening and selective alendronate for osteoporosis | 14.95 | 22.42 | | 3242.85 | 7.0657 | 17193 |
| Annual screening and selective alendronate for osteoporosis | 12.76 | 19.57 | | 4033.82 | 7.0783 | 37029 |
| One-time screening and selective alendronate for osteopenia | 13.48 | 20.30 | | 3875.66 | 7.0759 | 34413 |
| Annual screening and selective alendronate for osteopenia | 12.34 | 18.73 | | 4270.15 | 7.0821 | 39951 |
| *Age 70-74 years* |  |  | |  |  |  |
| No intervention | 23.91 | 34.91 | | 2993.11 | 5.7519 | Ref |
| Alendronate therapy without screening | 12.91 | 21.97 | | 3851.32 | 5.7933 | 20723 |
| One-time screening and selective alendronate for osteoporosis | 18.80 | 28.97 | | 3312.38 | 5.7734 | 14829 |
| Annual screening and selective alendronate for osteoporosis | 17.39 | 27.05 | | 3869.08 | 5.7803 | 30792 |
| One-time screening and selective alendronate for osteopenia | 16.69 | 25.92 | | 3951.40 | 5.7844 | 29475 |
| Annual screening and selective alendronate for osteopenia | 16.41 | 25.47 | | 4112.97 | 5.7854 | 33431 |
| *Age 75-79 years* |  |  | |  |  |  |
| No intervention | 29.12 | 43.78 | | 2797.74 | 4.4365 | Ref |
| Alendronate therapy without screening | 15.68 | 27.74 | | 3499.82 | 4.4742 | 18642 |
| One-time screening and selective alendronate for osteoporosis | 22.68 | 36.24 | | 3091.65 | 4.4529 | 17892 |
| Annual screening and selective alendronate for osteoporosis | 19.91 | 32.52 | | 3531.85 | 4.4604 | 30725 |
| One-time screening and selective alendronate for osteopenia | 20.33 | 32.63 | | 3622.07 | 4.4628 | 31382 |
| Annual screening and selective alendronate for osteopenia | 19.80 | 31.78 | | 3742.69 | 4.4643 | 34003 |

MOF: major osteoporotic fracture; All: all type fragility fractures; QALY: quality-adjusted life-year; ICER: incremental cost-effectiveness ratio.

**Table S5.** Outcomes of each intervention strategy including additional screening interval intervention at age 60-64 years

| Interventions |  |  | Incremental | |  |
| --- | --- | --- | --- | --- | --- |
|  | Costs, $ | QALYs | Costs, $ | QALYs | ICER, $/QALY |
| No intervention | 2832.43 | 8.2667 | Ref | Ref | Ref |
| Alendronate therapy without screening | 4064.79 | 8.2987 | 1232.36 | 0.0320 | 38471 |
| One-time screening and selective alendronate for osteoporosis | 3106.48 | 8.2824 | 274.05 | 0.0158 | 17368 |
| Annual screening and selective alendronate for osteoporosis | 4001.57 | 8.2921 | 1169.14 | 0.0254 | 46007 |
| Screening per two years and selective alendronate for osteoporosis | 3975.40 | 8.2920 | 1142.97 | 0.0254 | 45068 |
| Screening per five years and selective alendronate for osteoporosis | 3950.79 | 8.2919 | 1118.357 | 0.0253 | 44283 |
| One-time screening and selective alendronate for osteopenia | 3829.39 | 8.2914 | 996.96 | 0.0248 | 40266 |
| Annual screening and selective alendronate for osteopenia | 4289.83 | 8.2942 | 1457.40 | 0.0275 | 52911 |
| Screening per two years and selective alendronate for osteopenia | 4286.72 | 8.2942 | 1454.29 | 0.0275 | 52808 |
| Screening per five years and selective alendronate for osteopenia | 4280.26 | 8.2942 | 1447.83 | 0.0275 | 52614 |

**Table S6.** Outcomes of each intervention strategy in changing treatment adherence at age 60-64 years

| Interventions |  |  | Incremental | |  |
| --- | --- | --- | --- | --- | --- |
|  | Costs, $ | QALYs | Costs, $ | QALYs | ICER, $/QALY |
| No intervention | 2832.43 | 8.2667 | Ref | Ref | Ref |
| Adherence decreased to 72% |  |  |  |  |  |
| Alendronate therapy without screening | 4192.92 | 8.2898 | 1360.49 | 0.0231 | 58830 |
| One-time screening and selective alendronate for osteoporosis | 3180.18 | 8.2789 | 347.74 | 0.0123 | 28342 |
| Annual screening and selective alendronate for osteoporosis | 4122.09 | 8.2858 | 1289.66 | 0.0191 | 67500 |
| One-time screening and selective alendronate for osteopenia | 3933.13 | 8.2856 | 1100.69 | 0.0189 | 58170 |
| Annual screening and selective alendronate for osteopenia | 4408.89 | 8.2875 | 1576.46 | 0.0208 | 75627 |
| Adherence decreased to 54% |  |  |  |  |  |
| Alendronate therapy without screening | 4264.99 | 8.2852 | 1432.55 | 0.0185 | 77256 |
| One-time screening and selective alendronate for osteoporosis | 3218.31 | 8.2770 | 385.87 | 0.0103 | 37284 |
| Annual screening and selective alendronate for osteoporosis | 4192.59 | 8.2824 | 1360.16 | 0.0157 | 86567 |
| One-time screening and selective alendronate for osteopenia | 3995.19 | 8.2823 | 1162.76 | 0.0157 | 74287 |
| Annual screening and selective alendronate for osteopenia | 4478.68 | 8.2838 | 1646.25 | 0.0172 | 95781 |

**Table S7.** Outcomes of each intervention strategy in changing therapeutic drugs at aged 60-64 years

| Interventions |  |  | Incremental | |  |
| --- | --- | --- | --- | --- | --- |
|  | Costs, $ | QALYs | Costs, $ | QALYs | ICER, $/QALY |
| No intervention | 2832.43 | 8.2667 | Ref | Ref | Ref |
| Denosumab as therapeutic drug |  |  |  |  |  |
| **Denosumab** therapy without screening | 3310.55 | 8.2940 | 478.12 | 0.0273 | 17516 |
| One-time screening and selective **denosumab** for osteoporosis | 2930.93 | 8.2860 | 98.49 | 0.0194 | 5090 |
| Annual screening and selective **denosumab** for osteoporosis | 3604.04 | 8.2976 | 771.61 | 0.0310 | 24917 |
| One-time screening and selective **denosumab** for osteopenia | 3237.51 | 8.2960 | 405.08 | 0.0294 | 13785 |
| Annual screening and selective **denosumab** for osteopenia | 3497.84 | 8.2992 | 665.41 | 0.0326 | 20432 |
| Zoledronate as therapeutic drug |  |  |  |  |  |
| **Zoledronate** therapy without screening | 2737.76 | 8.2932 | -94.67 | 0.0265 | -3574 |
| One-time screening and selective **zoledronate** for osteoporosis | 2819.46 | 8.2793 | -12.97 | 0.0126 | -1028 |
| Annual screening and selective **zoledronate** for osteoporosis | 3326.91 | 8.2877 | 494.48 | 0.0210 | 23552 |
| One-time screening and selective **zoledronate** for osteopenia | 2835.42 | 8.2856 | 2.98 | 0.0190 | 157 |
| Annual screening and selective **zoledronate** for osteopenia | 2947.78 | 8.2878 | 115.35 | 0.0211 | 5457 |

**Table S8.** Outcomes of each intervention strategy including indirect costs at age 60-64 years

| Interventions |  |  | Incremental | |  |
| --- | --- | --- | --- | --- | --- |
|  | Costs, $ | QALYs | Costs, $ | QALYs | ICER, $/QALY |
| No intervention | 3181.93 | 8.2667 | Ref | Ref | Ref |
| Alendronate therapy without screening | 4366.51 | 8.2987 | 1184.59 | 0.0320 | 36980 |
| One-time screening and selective alendronate for osteoporosis | 3642.57 | 8.2824 | 460.64 | 0.0158 | 29193 |
| Annual screening and selective alendronate for osteoporosis | 4304.99 | 8.2921 | 1123.06 | 0.0254 | 44193 |
| One-time screening and selective alendronate for osteopenia | 4352.41 | 8.2914 | 1170.48 | 0.0248 | 47274 |
| Annual screening and selective alendronate for osteopenia | 4604.70 | 8.2942 | 1422.78 | 0.0275 | 51654 |

**Table S9**. Outcomes of each intervention strategy in 10-year horizon at age 60-64 years

| Interventions |  |  | Incremental | |  |
| --- | --- | --- | --- | --- | --- |
|  | Costs, $ | QALYs | Costs, $ | QALYs | ICER, $/QALY |
| No intervention | 500.79 | 5.4721 | Ref | Ref | Ref |
| Alendronate therapy without screening | 1970.82 | 5.4918 | 1470.04 | 0.0197 | 74697 |
| One-time screening and selective alendronate for osteoporosis | 802.54 | 5.4774 | 301.75 | 0.0053 | 56749 |
| Annual screening and selective alendronate for osteoporosis | 1574.76 | 5.4806 | 1073.97 | 0.0086 | 125440 |
| One-time screening and selective alendronate for osteopenia | 1649.17 | 5.4852 | 1148.39 | 0.0131 | 87351 |
| Annual screening and selective alendronate for osteopenia | 2150.36 | 5.4872 | 1649.57 | 0.0152 | 108758 |

**Reference**

1. Aromatase inhibitors versus tamoxifen in early breast cancer: patient-level meta-analysis of the randomised trials. The Lancet. 2015;386:1341–52.

2. Gao JJ, Cheng J, Bloomquist E, Sanchez J, Wedam SB, Singh H, et al. CDK4/6 inhibitor treatment for patients with hormone receptor-positive, HER2-negative, advanced or metastatic breast cancer: a US Food and Drug Administration pooled analysis. Lancet Oncol. 2020;21:250–60.

3. Wang L, Yu W, Yin X, Cui L, Tang S, Jiang N, et al. Prevalence of Osteoporosis and Fracture in China: The China Osteoporosis Prevalence Study. JAMA Netw Open. 2021;4:e2121106.

4. Mugnier B, Goncalves A, Daumas A, Couderc A-L, Mezni E, Viret F, et al. Prevention of aromatase inhibitor–induced bone loss with anti-resorptive therapy in post-menopausal women with early-stage breast cancer. Osteoporos Int. 2023;34:703–11.

5. Kanis JA, Johnell O, De Laet C, Johansson H, Oden A, Delmas P, et al. A meta-analysis of previous fracture and subsequent fracture risk. Bone. 2004;35:375–82.

6. Vandenput L, Johansson H, McCloskey EV, Liu E, Schini M, Åkesson KE, et al. A meta-analysis of previous falls and subsequent fracture risk in cohort studies. Osteoporos Int. 2024;35:469–94.

7. Yoshimura M, Moriwaki K, Noto S, Takiguchi T. A model-based cost-effectiveness analysis of osteoporosis screening and treatment strategy for postmenopausal Japanese women. Osteoporosis International. 2017;28:643–52.

8. Murad MH, Drake MT, Mullan RJ, Mauck KF, Stuart LM, Lane MA, et al. Clinical review. Comparative effectiveness of drug treatments to prevent fragility fractures: a systematic review and network meta-analysis. J Clin Endocrinol Metab. 2012;97:1871–80.

9. Qu B, Ma Y, Yan M, Wu H-H, Fan L, Liao D-F, et al. The economic burden of fracture patients with osteoporosis in western China. Osteoporosis international. 2014;25:1853–60.

10. National Healthcare Security Administration. http://www.nhsa.gov.cn/. Accessed 9 Oct 2023.

11. Wang Y, Cui H, Zhang D, Zhang P. Hospitalisation cost analysis on hip fracture in China: a multicentre study among 73 tertiary hospitals. BMJ Open. 2018;8:e019147.

12. Yang D, Zhang Y, Ma X, Huo L, Li L, Gao Y. Resources utilisation and economic burden of percutaneous vertebroplasty or percutaneous kyphoplasty for treatment of osteoporotic vertebral compression fractures in China: a retrospective claim database study. BMC Musculoskelet Disord. 2020;21:255.

13. National Bureau of Statistics. http://www.stats.gov.cn/. Accessed 9 Oct 2023.

14. Si L, Shi L, Chen M, Palmer AJ. Establishing benchmark EQ-5D-3L population health state utilities and identifying their correlates in Gansu Province, China. Quality of Life Research. 2017;26:3049–58.

15. Li S, Wang M, Liu L, Chen G. Which approach is better in eliciting health state utilities from breast cancer patients? Evidence from mainland China. European Journal of Cancer Care. 2019;28:e12965.

16. Chou T-C, Chiang S-C, Ko Y. Health state utilities for metastatic breast cancer in Taiwan. The Breast. 2020;51:57–64.

17. Si L, Winzenberg T, de Graaff B, Palmer AJ. A systematic review and meta-analysis of utility-based quality of life for osteoporosis-related conditions. Osteoporosis international. 2014;25:1987–97.

18. Hiligsmann M, Ethgen O, Richy F, Reginster J-Y. Utility values associated with osteoporotic fracture: a systematic review of the literature. Calcified tissue international. 2008;82:288–92.

19. Liu N, Zeng L, Li Z, Wang J. Health-related quality of life and long-term care needs among elderly individuals living alone: a cross-sectional study in rural areas of Shaanxi Province, China. BMC Public Health. 2013;13:313.

20. Zhang C, Feng J, Wang S, Gao P, Xu L, Zhu J, et al. Incidence of and trends in hip fracture among adults in urban China: A nationwide retrospective cohort study. PLoS Med. 2020;17:e1003180.

21. Lau EM, Woo J, Leung PC, Swaminthan R. Low bone mineral density, grip strength and skinfold thickness are important risk factors for hip fracture in Hong Kong Chinese. Osteoporos Int. 1993;3:66–70.

22. Bow CH, Cheung E, Cheung CL, Xiao SM, Loong C, Soong C, et al. Ethnic difference of clinical vertebral fracture risk. Osteoporos Int. 2012;23:879–85.

23. Kwok AWL, Gong J-S, Wang Y-XJ, Leung JCS, Kwok T, Griffith JF, et al. Prevalence and risk factors of radiographic vertebral fractures in elderly Chinese men and women: results of Mr. OS (Hong Kong) and Ms. OS (Hong Kong) studies. Osteoporos Int. 2013;24:877–85.

24. Tsang SWY, Kung AWC, Kanis JA, Johansson H, Oden A. Ten-year fracture probability in Hong Kong Southern Chinese according to age and BMD femoral neck T-scores. Osteoporos Int. 2009;20:1939–45.

25. Kwok TCY, Su Y, Khoo CC, Leung J, Kwok A, Orwoll E, et al. Predictors of non-vertebral fracture in older Chinese males and females: Mr. OS and Ms. OS (Hong Kong). J Bone Miner Metab. 2017;35:330–7.

26. Koh GC-H, Tai BC, Ang L-W, Heng D, Yuan J-M, Koh W-P. All-cause and cause-specific mortality after hip fracture among Chinese women and men: the Singapore Chinese Health Study. Osteoporos Int. 2013;24:1981–9.
